# Moving Women Veterans’ Health Research Forward: a Special Supplement

**DOI:** 10.1007/s11606-022-07606-1

**Published:** 2022-08-30

**Authors:** Alison B. Hamilton, Eleanor Bimla Schwarz, Holly N. Thomas, Karen M. Goldstein

**Affiliations:** 1 VA HSR&D Center for the Study of Healthcare Innovation, Implementation and Policy, VA Greater Los Angeles Healthcare System, Los Angeles, CA USA; 2 Department of Psychiatry and Biobehavioral Sciences, University of California (UC) Los Angeles, David Geffen School of Medicine, Los Angeles, CA USA; 3 Division of General Internal Medicine, UC San Francisco, Zuckerberg San Francisco General Hospital, San Francisco, CA USA; 4 Departments of Medicine and Clinical and Translational Research, University of Pittsburgh, Pittsburgh, USA; 5 VA HSR&D Center of Innovation to Accelerate Discovery and Practice Transformation, Durham VA Health Care System, Durham, NC USA; 6 Department of Medicine, Duke University School of Medicine, Durham, NC USA

Women’s increased rate of participation in the US military is reshaping the Veteran population. With the growing number of women Veterans, it is imperative to understand the unique facets of military women’s and women Veterans’ health and health care experiences to ensure that they receive the highest-quality patient-centered care throughout the life course. This special issue of the *Journal of General Internal Medicine* (JGIM), sponsored by VA Health Services Research & Development (HSR&D) Service and the VA Women’s Health Research Network, highlights innovations and new findings related to women Veterans’ health and health care, including the diverse needs and experiences of women Veterans and active-duty and Reserve/National Guard servicewomen. We received an extraordinary number of submissions, reflecting the richness and depth of the field. After careful review and reflection, we accepted papers clustered around the five themes described below.

## Sexual and Reproductive Health

The scientific literature about sexual and reproductive health among women Veterans is a relatively new field, which has largely been descriptive to date. This supplement includes nine papers that move this field forward, adding nuance and actionable findings. Monteith et al. report on the prevalence of firearm access among women Veterans of reproductive age and the need for safe firearm storage initiatives. Keddem et al. note underutilization of gonorrhea and chlamydia testing and higher rates of infection among young women Veteran VA users. Mattocks et al. explore beliefs about and low receipt of the COVID-19 vaccine by pregnant Veterans. Ahuja et al. provide an example of how VA facilities can improve care for pregnant Veterans through a VA facility quality improvement project which, after assessing primary care provider comfort with caring for pregnant Veterans, led to a new local education series. Friedman et al. note that nearly one in ten women Veteran VA primary care patients had no local VA gynecologist and inadequate county-level gynecologist supply.

Additional papers help us understand in more detail women Veterans’ family planning needs. Gawron et al. describe the low frequency of emergency contraception provision among women Veterans, while Callegari et al. report that women Veterans’ perceptions of contraception counseling vary by clinical setting in which the counseling was received. Judge-Golden et al. describe Veterans’ experiences with abortion and associations with mental health diagnoses, while Grindlay et al. contribute findings indicating that while unintended pregnancy rates are higher among active-duty US Navy members than among civilians, abortion rates appear to be lower.

## Sexual Violence

While the prevalence and burden of sexual trauma are well-described among women Veterans, this special issue includes three papers that set a foundation for future work in this area. Galovski et al. provide a narrative review of the state of the knowledge of VA military sexual trauma (MST) research, including prevalence compared to men, common mental health sequelae, and associated adverse health outcomes. Iverson et al. help us understand the impact of the COVID-19 pandemic on experiences of IPV and associations with post-traumatic stress disorder (PTSD) and depressive symptoms. Hollis et al. advance methods of identifying experiences of sexual violence through using multiple data sources from the medical record.

## Mental Health

Mental health has long been a primary focus of women Veterans’ health research; the work in this supplement highlights new advances in this field. These papers highlight ongoing needs among subpopulations of women Veterans, explore opportunities for delivery of care, and test new interventions designed for women Veterans. Gender comparisons regarding MST detection for MST detection (McLean et al.) and openness to eating disorder screenings (Hardin et al.) help us understand whether and when women may need gender-specific mental health care. Included papers also push us to consider areas in which women Veterans’ mental health care could be improved, including polypharmacy of psychoactive medications and associated suicide risks and unintended overdose death (Gibson et al.), the potential mismatch between the desire for and use of mental health services during pregnancy (Kroll-Desrosier et al.), and preferred modalities such as video visits for women’s mental health care (Lindsay et al.). This supplement also includes a report of a randomized trial by Saban et al. which compared mindfulness-based stress reduction (MBSR) to an active control among women Veterans with risk factors for cardiovascular disease and found that those who participated in MBSR reported less perceived stress, loneliness, and symptoms of PTSD.

## Women Veterans’ Health Needs

As our understanding of women Veterans’ health conditions and needs evolves, we must grow our knowledge of how to structure the health care workforce and what areas of clinical care must be improved to address ongoing disparities in quality of care. To this end, we include four studies that elucidate the complex health issues faced by women Veterans and the areas needing improvement among those caring for this prioritized population. Lehavot et al. identified the unique health care needs of women Veterans with lower-extremity amputation, and Dhruva et al. identify persistent gender-based disparities in guideline-directed medical therapy among younger women Veterans. Sheehan et al. describe the significant multimorbid physical conditions, mental health diagnoses, and trauma histories of women Veterans using VA primary care, while Farkas et al. explore how VA women’s health primary care providers can prepare for and sustain their work in caring for the medical complexity of women Veterans by receiving training at women’s health mini-residencies.

## Innovations in Clinical Practice

Finally, we included three papers that round out the supplement by describing efforts to improve the way that care is delivered to women Veterans. Galovski et al. describe WoVeN, a unique national social network of women Veterans that trains peer leaders in facilitating group sessions with women Veterans. Miller et al. describe the innovative national reproductive mental health consultation program and evaluation. Spinella et al. describe an integrated multidisciplinary women Veteran substance use disorder primary care clinic.

Collectively, this outstanding set of papers builds on the research agenda laid out over a decade ago by Yano and colleagues^1^; that agenda remains relevant today and reflects the ongoing health concerns of many women Veterans.^1^ The work included in this supplement advances the field by offering a deeper understanding of women Veterans’ and active-duty military women’s health and health care needs. In a 2017 evidence map of the women Veterans’ health research literature,^2^ the majority of the papers in the field were observational studies, suggesting an opportunity for more interventional and implementation science.^1^ This supplement provides examples of how VA women’s health research continues to develop with thoughtful nurturing and support, both to expand the types of study designs employed and to include a focus on the range of health needs of this population (see Yano and Tomoyasu editorial, this issue).

We acknowledge the limitations of language focused on “women’s health,” recognizing the gender diversity of Veterans and that “cisgender women are not the only ones who need ‘women’s health’ care and research.”^3^ We agree with Boerner and colleagues, who suggest that researchers should be “conceptualizing gender as a more fluid construct that can shift with changes in hormones, abilities, roles, and socially imposed expectations…”^4^ As our understanding of VA users begins to better reflect the reality of the diversity of Veteran gender identity, it will be critical not only to conceptualize and address gender more inclusively, but also other intersecting identities.^5^ We continue to need to identify when gender-tailoring of care is or is not necessary, and how to effectively intervene upon persistent disparities (e.g., in cardiovascular disease^6^) and how to prevent new disparities from developing. We must learn from our experience during a world-changing, multi-year health crisis, and pursue research that addresses the “external context,” or the forces and influences surrounding women’s lives and health care (e.g., environmental exposures, restricted access to critical reproductive health care, climate change^7^) that will affect their social roles, personal well-being, and family structure. Finally, we will be interested to see how VA women’s health care evolves in response to recent congressional mandates for increased access to and satisfaction with VA care, for example, via efforts to provide child care and to eliminate harassment on VA grounds.^8^

This collection of cutting-edge research in women’s health will be of interest both within and beyond the VA health care system. This work is relevant not only to Veterans and VA employees, but also to women’s health clinicians outside of the VA as many health conditions (e.g., PTSD secondary to sexual trauma, complex co-morbid diagnoses) and health care challenges (e.g., barriers to reproductive health care, disparities in guideline-concordant cardiovascular disease care) that are prevalent among our Veterans are common among civilian women populations as well. We appreciate VA Health Services Research & Development for providing this opportunity to disseminate exemplary women’s health research, and the *Journal of General Internal Medicine* for supporting this special issue and providing a vibrant home for VA health services research.
